# Discovery of Anti-Neuroinflammatory Compounds in Alzheimer’s Disease: Current Trends and Future Perspectives

**DOI:** 10.1007/s12035-026-06114-1

**Published:** 2026-08-08

**Authors:** Nantaphat Paelaong, Kittisak Sripha, Yingrak Boondam

**Affiliations:** 1 Faculty of Pharmacy, Mahidol University, Si Ayutthaya Rd, Bangkok, 10400 Thailand; 2 Department of Pharmaceutical Chemistry, Faculty of Pharmacy, Mahidol University, Si Ayutthaya Rd, Bangkok, 10400 Thailand; 3 Department of Physiology, Faculty of Pharmacy, Mahidol University, Si Ayutthaya Rd, Bangkok, 10400 Thailand; 4 Centre of Biopharmaceutical Science for Healthy Ageing, Faculty of Pharmacy, Mahidol University, Bangkok, 10400 Thailand; 5 Unit of Compound Library for Drug Discovery, Mahidol University, Bangkok, 10400 Thailand

**Keywords:** Neuroinflammation, Drug discovery, Alzheimer’s disease, Blood–brain barrier, Chemical synthesis

## Abstract

**Abstract:**

Chronic neuroinflammation in response to abnormal protein aggregates has been implicated as a critical mechanism in several neurodegenerative diseases, and the modulation of signaling pathways associated with inflammation is being explored as a potential strategy for new therapeutics. In Alzheimer’s disease (AD), the neuroinflammation hypothesis also starts to influence the landscape of drug discovery in place of the previously regarded amyloid hypothesis. As such, the objective of this review is to compile recent advanced in the discovery of anti-neuroinflammatory compounds developed through chemical synthesis and structural modification, providing an overview of current trends in drug discovery strategies and scaffold redesign for the development of the next-generation of anti-neuroinflammatory therapeutics. It was found that the compounds described in the publications could be categorized into four groups: modification of natural compounds, rational drug design, drug screening approaches, and drug repurposing. While the categorization seems to be similar to drug discoveries in other fields, with the modification of natural compounds accounting for most publications, specific observations could still be made. Namely, the growing interest in the multitarget-directed ligand approach reflects the increasing recognition of AD as a multifactorial disease. Despite this, further research is required to identify additional effective activities or the appropriate combinations besides anti-neuroinflammatory activity. Lastly, this review also gives brief overview regarding physicochemical properties of central nervous system–active agents, of which anti-neuroinflammatory compounds are parts of, and suggests the adoption of in silico prediction models as screening tools before the use of in vitro or in vivo models.

**Graphical Abstract:**

(Created with the program Inkscape. The brain icon illustration was taken from NIAID NIH BioArt Source (bioart.niaid.nih.gov/bioart/60))

**Figure:**
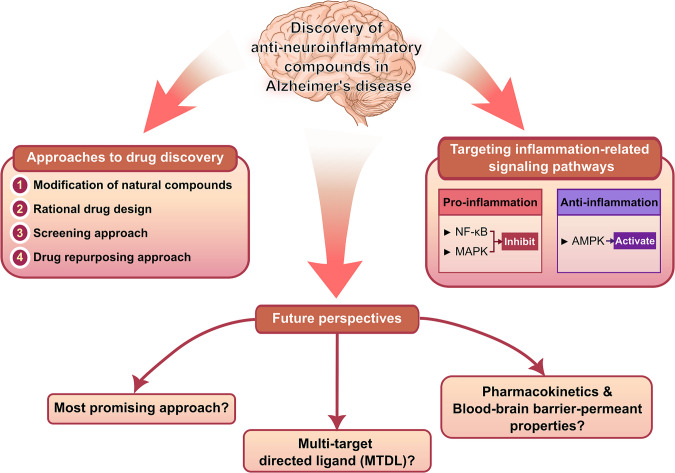

## Introduction

### Neuroinflammation and Neurodegenerative Diseases

Neuroinflammation is an inflammatory response in the presence of pathogens or harmful substances within the central nervous system (CNS). This response is mediated by glial cells like microglia and astrocytes [1]. In short terms, neuroinflammation serves as a defense mechanism of the CNS, in which pathogenic substances are eliminated by several mechanisms including microglial phagocytosis, oxidative stress, and degrading enzymes. However, chronic and persistent inflammation can lead to the excessive release of proinflammatory mediators and free radicals, resulting in neuronal damage [1–3]. Factors contributing to chronic inflammation include endogenous factors, such as protein aggregates and genetic mutations, and exogenous factors, such as systemic infection, trauma, gut microbiota dysbiosis, and aging [1, 2]. Notably, the existence of protein aggregates that trigger chronic neuroinflammation is a shared characteristic in many neurodegenerative diseases [1, 4].

Neurodegenerative diseases are a group of disorders characterized by the loss of neurons in terms of both quantity and function. The examples of these diseases are Alzheimer’s diseases (AD), Parkinson’s disease (PD), amyotrophic lateral sclerosis (ALS), prion diseases, and Huntington’s disease [5]. It was suggested that neuronal loss occurring in these diseases stems from similar mechanisms: persistent neuroinflammation induced by the deposition of pathogenic protein within the brain. Chronically activated glial cells not only have reduced phagocytic capability, but also they might be responsible for neuronal damage and the exacerbation of pathogenic protein aggregation. Moreover, cell debris from damaged neurons can activate glial cells into releasing even more proinflammatory, neurotoxic mediators, forming a vicious cycle [1, 2, 4, 6]. Therefore, neuroinflammation is considered one of the key factors influencing the development and progression of these diseases.

### Roles of Microglia in Neuroinflammation

Microglia are one of the glial cells responsible for supporting neurons. In particular, microglia serve as the resident immune cells of the brain and have important roles in maintaining homeostasis [1, 6]. In a healthy brain, microglia exist in the resting state, mainly performing immunosurveillance and housekeeping roles, such as synaptic pruning [7, 8]. However, in response to threats including pathogens, injuries, or harmful substances, they could undergo a process called microglial activation into either M1 (classical) or M2 (alternative) phenotypes [6, 9]. The M1 phenotype is generally considered the proinflammatory phenotype, as it produces inflammatory mediators such as tumor necrosis factor-α (TNF-α), interleukin (IL)−1β, IL-6, CC chemokine ligand (CCL) 2, nitric oxide (NO), and reactive oxygen species (ROS), all of which contribute to neuronal damage through various mechanisms including apoptosis induction and oxidative stress. In contrast, the M2 phenotype is associated with neuroprotection. This is due to its role in resolving inflammation and promoting tissue repair through the release of anti-inflammatory mediators such as IL-10, transforming growth factor (TGF)-β), found in inflammatory zone 1 (FIZZ1), and arginase 1 (Arg1) [9, 10]. Despite this, the activation of microglia into either phenotype is not set in stone, as the M1 phenotype can polarize into M2, depending on several modulators [9].

Several substances can initiate microglial activation and promote polarization into the M1 phenotype. For example, pathogen-associated molecular patterns (PAMPs) are molecules released by pathogenic organisms, such as lipopolysaccharide (LPS), from the cell wall of gram-negative bacteria, and viral genetic materials. Similarly, damage-associated molecular patterns (DAMPs) are endogenous molecules originating from the cytoplasm or nucleus that get released by damaged or dying cells; examples include heat shock proteins (HSPs) and ATP [11, 12]. Both PAMPs and DAMPs bind to specific pattern recognition receptors (PRRs) on the surface of microglia, triggering downstream signaling pathways that lead to the release of proinflammatory mediators in response to such threats [9, 13]. Microglial activation involves many signaling pathways that regulate both proinflammatory response and the resolution of inflammation. These pathways include but are not limited to nuclear factor-κB (NF-κB), mitogen-activated protein kinases (MAPKs), and adenosine monophosphate–activated protein kinase (AMPK). Below, a brief overview of each pathway is provided (for their more in-depth reviews, please see [14–18]).

NF-κB is a family of inducible transcription factors whose primary role in microglia is the regulation of inflammatory gene expression [14]. Under normal condition, NF-κB is bound to a family of inhibitory proteins, mainly IκBα, within the cytoplasm, and it requires upstream signaling for the activation. Particularly, in the canonical pathway, IκBα is phosphorylated, marking it for ubiquitin-dependent degradation. This process is generally associated with the activation of Toll-like receptor (TLR), one of the PRRs, and subsequent downstream kinases. Once free from the IκBα, NF-κB translocates to the nucleus, where it binds to DNA to regulate the expression of proinflammatory mediators. These mediators consist of cytokines like TNF-α and IL-6 and enzymes such as cyclooxygenase (COX)−2 and inducible nitric oxide synthase (iNOS) which produces prostaglandin (PG) and NO, respectively [9, 14, 15]. Due to the importance of NF-κB in inflammatory signaling pathways, several NF-κB inhibitors have been investigated as potential anti-neuroinflammatory agents [6, 14].

MAPKs are a family of intracellular serine/threonine kinases involved in diverse cellular processes, including cell proliferation, differentiation, development, inflammation, and apoptosis. The MAPK family is categorized into three subgroups: extracellular signal-regulated kinases 1/2 (ERK1/2), p38 MAPK, and c-Jun N-terminal kinases (JNK) [19, 20]. It has been firmly established that p38 and JNK, which can be activated by various stressors including proinflammatory cytokines and ROS, play a major role in neuroinflammation as well as the pathogenesis of various neurodegenerative disease via the phosphorylation of pro-inflammatory transcription factors or modulation of other pathways like NF-κB [16]. In contrast, the role of ERK1/2 is less clear due to conflicting evidence indicating both pro- and anti-inflammatory activities. On the one hand, ERK1/2 was reported to be a regulator of NF-κB activation in a similar manner to p38 and JNK. On the other hand, ERK1/2 seems to have a neuroprotective effect following prion infection in animal models. In addition, ERK1/2 could also increase the activation of nuclear factor erythroid 2–related factor 2 (Nrf2) which regulates the expression of several antioxidant enzymes [21, 22].

AMPK is a serine/threonine kinase that play a critical role in regulating energy metabolism. Through either binding with its ligand, adenosine monophosphate (AMP), or being phosphorylated by upstream kinases, activated AMPK could inhibit anabolism and promote catabolism during an energy-deficient state [17, 23]. In addition, AMPK is also a modulator of inflammation, as it could indirectly inhibit NF-κB activation through several pathways, including sirtuin 1 (SIRT1), forkhead box O (FOXO) transcription factors, and peroxisome proliferator–activated receptors (PPARs). Similarly, activation of AMPK is associated with antioxidant activities due to its ability to further activate Nrf2, the transcription factor of antioxidant enzymes [24, 25].

To summarize, microglia are the resident immune cells of the CNS. In the chronically activated state, they release proinflammatory mediators that contribute to neuroinflammation and the subsequent development of neurodegenerative diseases. Several signaling pathways are involved in regulating microglial activity, including proinflammatory pathways such as NF-κB and MAPK and anti-inflammatory pathways such as AMPK. As such, modulating these pathways may represent a promising therapeutic strategy for the treatment of neurodegenerative diseases.

### Alzheimer’s Disease (AD) and Its Current Therapeutic Approaches

AD is a neurodegenerative disease which is the most common cause of dementia, accounting for 60–70% of all dementia cases [26]. It is characterized by progressive memory loss and cognitive impairments, which can eventually lead to complete dependency and death [27]. AD is classified into two types based on the age of onset. The first is the familial or early-onset AD, which manifests before the age of 65 and is associated with certain genetic mutations. On the other hand, the sporadic or late-onset AD occurs after the age of 65 and is linked to various risk factors, both genetic and environmental [28].

Anatomically, the brains of AD patients exhibit atrophy, especially in the hippocampus and cerebral cortex, indicating neuronal loss. On the microscopic scale, the pathological hallmarks of AD are extracellular beta amyloid (Aβ) plaques and intracellular neurofibrillary tangles (NFTs) [28, 29]. Aβ plaques are aggregates of Aβ monomers generated via the amyloidogenic pathway, in which amyloid precursor proteins (APPs) are sequentially cleaved by β-secretase and γ-secretase. These Aβ monomers, especially the predominant isoform with 42 amino acids (Aβ_1–42_), can form insoluble Aβ fibrils and plaques or soluble oligomers that spread throughout the brain [28, 30]. In contrast, tau proteins normally contribute to the assembly and stabilization of microtubules. However, when hyperphosphorylated, tau proteins can aggregate to form NFTs within neurons [31]. The accumulation of both Aβ plaques and NFTs is believed to play a role in neurotoxicity and synaptic loss, ultimately resulting in neuronal death and cognitive impairment [28].

Currently, several medications have been approved for the treatment of AD. Excluding brexpipazole, an oral antipsychotic which was approved for treatment of agitation associated with AD, other treatment options could be categorized into three classes based on their mechanisms of action [32, 33]. The first is the acetylcholinesterase inhibitors (AChEIs) which include donepezil, rivastigmine, and galantamine. The mechanism of AChEIs is the inhibition of acetylcholinesterase (AChE), resulting in reduced degradation and an increase in acetylcholine (ACh), a neurotransmitter that plays a crucial role in learning and memory. The next class of drugs is N-methyl-D-aspartate (NMDA) receptor antagonists, consisting solely of the drug memantine. Its mechanism involves amelioration of Ca^2+^-induced neurotoxicity resulting from NMDA receptor overactivation [28, 32, 34]. Both classes of drugs, administered either alone or in combination, could improve cognitive and behavioral symptoms of AD patients. However, they are only effective for a short time and cannot stop or delay the progression of the disease [32, 33]. In contrast to the previous two classes of small-molecule drugs, the most recent AD therapeutics, lecanemab and donanemab, are monoclonal antibodies (mAbs) that target Aβ peptides and plaques. These drugs were demonstrated to facilitate Aβ clearance and improve cognitive function of the patients in clinical trials leading to their approvals [32]. Nonetheless, anti-Aβ therapeutics are offset by several drawbacks. In addition to their higher cost compared to traditional AD therapy, they are also associated with amyloid-related imaging abnormalities (ARIA), abnormal magnetic resonance imaging (MRI) signals whose associated neurological symptoms require prompt assessment and care [32, 35]. Moreover, the recently published systematic review and meta-analysis on anti-Aβ mAbs did not find any incremental benefits in terms of cognitive function and dementia severity compared to the placebos, although the conclusions were based on very few eligible studies [36].

As reflected by the diverse classes of its therapeutics, multiple hypotheses have been proposed as the principal driver of AD pathogenesis and progression since the first description of the disease in 1906 [32, 37]. Some of these hypotheses have served as rationale behind the discovery and development of aforementioned therapeutics and many more currently undergoing preclinical investigation or clinical trials. In the following section, some of the more prominent hypotheses are briefly discussed in terms of both supporting evidence and counterarguments.

The cholinergic hypothesis was the first hypothesis proposed as the cause of AD [32]. As mentioned, ACh is a neurotransmitter that plays a crucial role in learning and memory. It was found that in the brain of AD patients, there is a severe loss of cholinergic neurons, particularly in the nucleus basalis of Meynert. Additionally, the activity of choline acetyltransferase (ChAT), the enzyme responsible for ACh synthesis, is significantly reduced in the remaining neurons. These findings, which closely correlate with the cognitive deficits and memory loss observed in AD patients, led to the establishment of the cholinergic hypothesis and subsequent development of AChEIs that could restore the ACh levels in the brain [38, 39]. Despite their short-term benefits of cognitive improvement, AChEIs are unable to alter the course of the disease, which led researchers to seek new hypotheses that could better explain AD pathogenesis. Even so, AChEIs remain the standard of care for AD treatment until today [38].

The amyloid hypothesis was the next widely regarded hypothesis. In essence, it is posited that the deposition of Aβ in the brain is the central event that drives the development of AD. Namely, it promotes both neurodegeneration and the formation of NFTs through several mechanisms [32, 40]. This hypothesis is supported by evidence from familial AD, where mutations in genes involved in Aβ processing, such as amyloid precursor protein (APP), presenilin 1 (PSEN1), and presenilin 2 (PSEN2), result in its overproduction. In addition, the apolipoprotein E (APOE) ε4 allele, one of the major genetic risk factors in sporadic AD, was found to impair Aβ clearance [32]. Therefore, therapeutic approaches according to this hypothesis is to reduce Aβ production or prevent its aggregation to delay disease progression. Both small-molecule and biologic drugs have been developed for such purposes, including β- and γ-secretase inhibitors, Aβ aggregation inhibitors, and anti-Aβ mAbs and vaccines [32, 34]. Despite this, several clinical trials of such drugs were discontinued due to lack of improvement or decline in the cognitive function of the subjects [32, 41]. Moreover, as mentioned, the benefit of anti-Aβ mAbs, including the already approved lecanemab and donanemab, over traditional treatment is still uncertain [36]. These outcomes prompted a reassessment of the hypothesis. While the fact that Aβ peptides are neurotoxic still holds true, it was discovered that substantial Aβ accumulation also occurs in the brain of cognitively healthy elderly. Furthermore, the spread of hyperphosphorylated tau correlates more closely with neurodegeneration compared to Aβ pathology in terms of both timeline and locations. These pieces of evidence suggests that the aggregation of Aβ alone does not necessarily lead to AD, and that other mechanisms are likely involved [42, 43].

The neuroinflammation hypothesis, which states that the sustained microglial activation and chronic inflammatory responses within the CNS play a critical role in the development of AD, emerged in recent years to explain the gap between Aβ pathology and AD [6, 32]. As mentioned, inflammation in response to pathogenic protein aggregates seems to be a common feature among neurodegenerative diseases. In AD, this is supported by the fact that several genetic risk factors associated with sporadic AD are related to immune functions or expressed exclusively by microglia [44]. In addition, the results from experimental studies suggested that microglial activation and the resultant release of proinflammatory mediators could exacerbate Aβ deposition and tau hyperphosphorylation, as well as contribute to neuronal damage. These findings were collaborated by the study on the brains of AD patients, in which microglia were found to colocalize and surround Aβ plaques as if acting as barriers [2, 6, 32, 37, 44]. As such, compounds that could attenuate neuroinflammation and prevent microglial activation have been proposed as a potential intervention strategy for AD. Previously, several observational studies have reported an inverse correlation between regular use of nonsteroidal anti-inflammatory drugs (NSAIDs) and the risk of AD development; however, randomized controlled trials have failed to demonstrate significant therapeutic benefits of NSAIDs in AD treatment [6, 45]. Despite this, several drugs targeting neuroinflammation and its related signaling pathways are currently under development, with some progressing into clinical trials [37].

As the focus in the field of AD drug discovery and development shifted from the amyloid hypothesis to neuroinflammation hypothesis, more and more anti-inflammatory compounds are now being investigated as potential AD therapeutics. While there have been several reviews regarding the neuroinflammatory activities of natural compounds [46–49], few provide a general overview of anti-neuroinflammatory compound discovery via chemical synthesis. Therefore, this review aims to compile literature on the discovery of such compounds to provide a comprehensive understanding the rationale behind their development, identify current trends in AD therapeutic research, and discuss prospective directions for the development of next-generation therapeutics.

## Discovery of Anti-Neuroinflammatory Compounds for the Treatment of AD

### Methodology

**Fig. 1:**
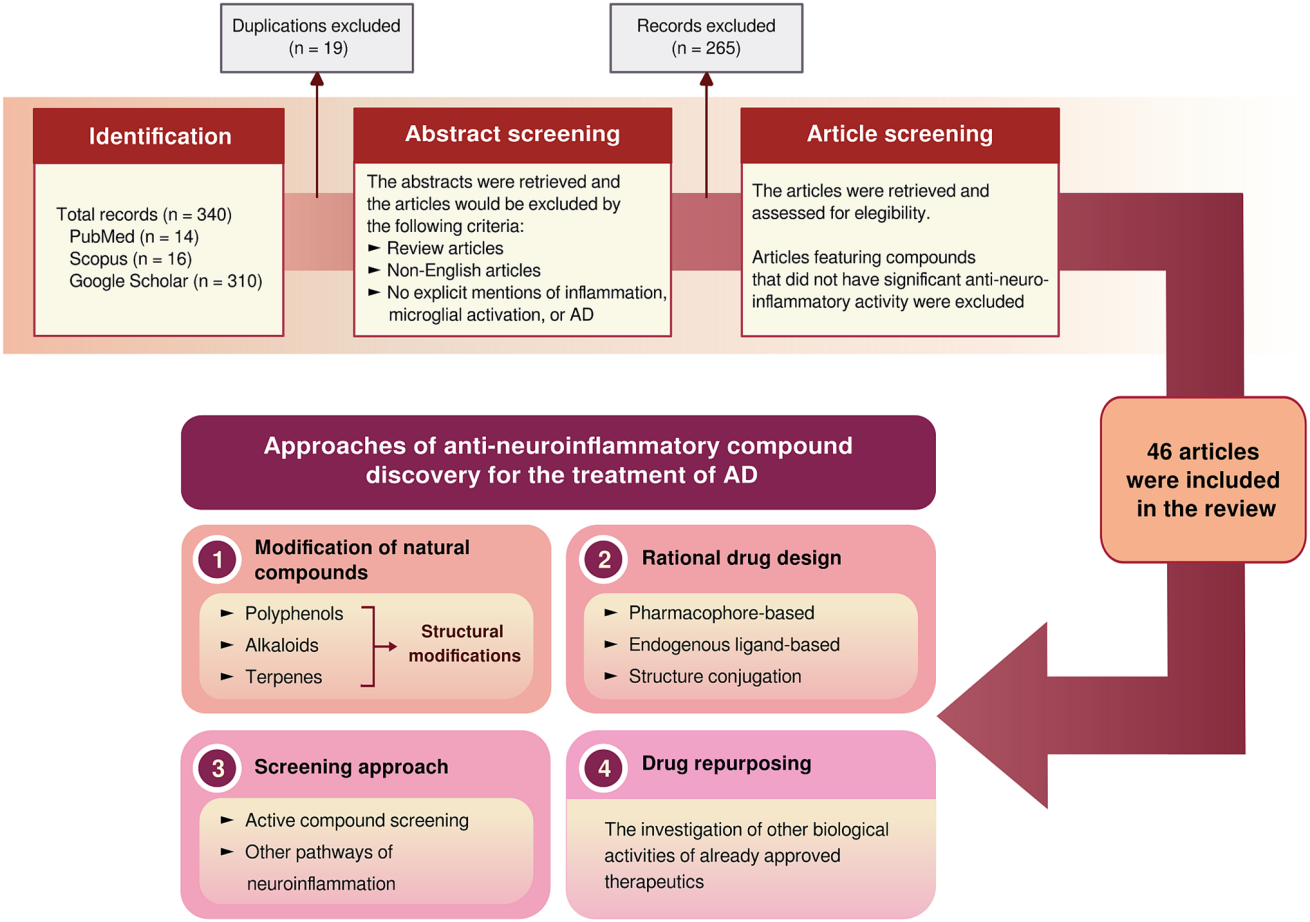
A brief overview on literature search methodology and selection

The objective of this review was to compile the publications involving the discovery of anti-neuroinflammatory compounds derived from chemical synthesis, specifically in the context of microglial activation in AD, in order to understand the current trends in the field and extrapolate on the future of AD drug discovery. As such, the literature search published between 2014 and 2025 was first conducted across three databases: PubMed, Scopus, and Google Scholar, using the combination of following queries: “anti”, “neuroinflamm*”, “inflamm*”, “microglia”, “BV-2”, “synthe*”, “compounds”, and *“Alzheimer’s disease”*. The retrieved publications were then subjected trough the screening processes as detailed in Fig. 1. Ultimately, 46 articles were included in this review. After categorization based on the approach to drug discovery, the findings from each article, with a focus on anti-inflammatory activities, were briefly summarized. The structures of all featured compounds were redrawn in accordance with their respective references using ACD/ChemSketch (Freeware) 2024.2.0 and attached along with the findings.

### Considerations Regarding the Permeability of Anti-Neuroinflammatory Compounds

Before proceeding to the discussion of current trends in AD drug discovery and development, it is important to stress that compounds that demonstrate remarkable effectiveness in preclinical research do not necessarily become viable drug candidates. For medications, especially small-molecule medications indicated for chronic diseases, oral absorption and bioavailability are important factors that contribute to increased patient compliance and accessibility. Presently, there are several methods to predict the intestinal permeability of drug candidates during the preclinical phases. Firstly, “drug-like properties” of a small molecule could be determined based on their molecular composition and structure using Lipinski’s rule of five, which states that that poor absorption or permeation is more likely when the molecules have more than five H-bond donors, a molecular weight (MW) over 500 Da, a calculated logarithm of partition coefficient (clogP) over 5, and more than 10 H-bond acceptors [50]. This rule, along with other models which have been incorporated in several in silico prediction tools, is beneficial for the selection of molecules during the preliminary screening process. For a more accurate prediction, both in vitro (e.g., Transwell assay using either MDCK or Caco-2 cell line) and in vivo assays have been employed to determine drug permeability in experimental settings [51].

Besides intestinal permeability, compounds with anti-neuroinflammatory activity still need to overcome another challenge to arrive at their target site. BBB, which is composed of capillary endothelium connected to one another by tight junctions, acts as a physical barrier between blood and the CNS. The original function of BBB is maintaining the ionic composition homeostasis within the CNS to ensure the reliability of electrochemical signal conduction; however, the heavily regulated barrier also serves as a protection against toxins and exogenous substances, which unfortunately includes therapeutic drugs [52]. Out of a large library of small-molecule drugs, only a few percent of drugs could cross the BBB. And from these molecules, two important observations were revealed: the MW of BBB-permeant molecules generally do not exceed 400–500 kDa and that they have no more than 10 hydrogen donors and acceptors combined [53]. Therefore, several BBB models, such as parallel artificial membrane permeability assay (PAMPA) and cell-based assays, have been developed as drug screening tools [54]. Even so, in vitro studies cannot fully predict BBB permeability of a compound in actual organisms. This is because they only account for the ability of compounds to pass through BBB through passive diffusion, when in reality, various BBB transporters could either further promote or, much more importantly, hinder their CNS uptake. Specifically, solute carrier (SLC) family of carrier-mediated transport and efflux pumps like ATP-dependent P-glycoprotein (P-gp) could affect the rate and extent at which compounds could pass through BBB [51–53]. As such, in vivo models, which can account for all forms of small-molecule transport, are still employed in later stages of drug development [51–53, 55]

### Current Trends in Anti-Neuroinflammatory Compound Discovery and Development for the Treatment of AD

From 46 eligible articles, anti-neuroinflammatory compounds were identified using both in vitro (primary or immortalized cell lines induced by LPS or Aβ) and in vivo (transgenic or LPS/Aβ-induced rodents) neuroinflammation models. In addition, patterns of anti-neuroinflammatory compound discovery among these articles were identified and categorized into four main groups based on their main approaches to drug discovery (Fig. 1): (1) modification of natural compounds, (2) rational drug design, (3) screening approaches, and (4) drug repurposing. The main findings of each study are included in the tables of their respective groups. On the other hand, in-text discussions are dedicated to the rationale behind each approach, the shared characteristics of studies included, as well as the examples of compounds, particularly ones with notable activities.

#### Modification of Natural Compounds

Natural products, including plants and animals, have been the sources of medicine since ancient times. Even today, several drugs in use originate from or are derived from nature [56]. Consequently, drug discovery from natural products remains a viable strategy. However, natural compounds often have structural limitations, such as pharmacologically irrelevant moieties, a lack of nitrogen or halogen atoms, and complex chirality. These limitations can interfere with pharmacological activities or contribute to the toxicities. Therefore, modifying natural compounds to enhance their activity, safety, pharmacokinetics, or physicochemical properties is beneficial in terms of drug discovery [57]. Furthermore, due to the available literature regarding the biological activities of the compounds, selecting compounds with already established biological activities to further improve upon is also a good strategy.

An example of the modification of natural compounds in the context of AD is the development of rivastigmine. Rivastigmine is a semisynthetic compound derived from physostigmine, which is a natural compound obtained from the plant *Physostigma venenosum*. Physostigmine was explored as a potential acetylcholinesterase inhibitor (AChEI) for the treatment of AD. However, its clinical use was limited by a lack of efficacy and severe side effects. To improve its activity and pharmacokinetics, as well as to reduce its toxicity, several semisynthetic derivatives were developed. Among them, rivastigmine is now considered a second-generation AChEI and remains a first-line treatment for AD, available in both oral and transdermal dosage forms [32, 58].

The following sections and Table 1 discuss the discovery of anti-neuroinflammatory compounds derived from the modification of natural compounds, categorized based on their structures [59–74].

**Table 1:**
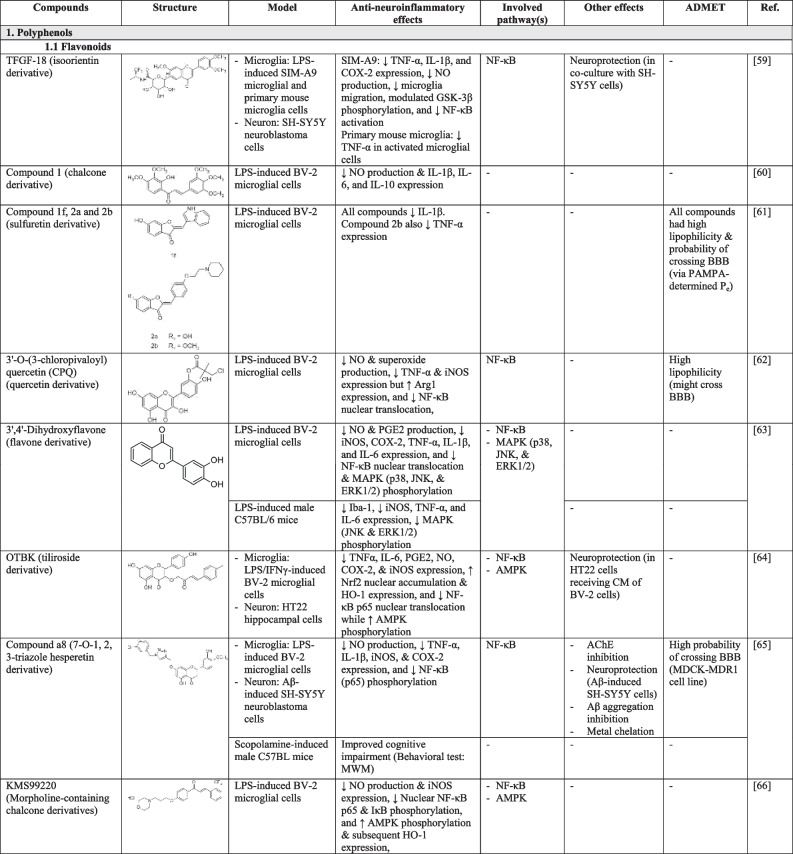

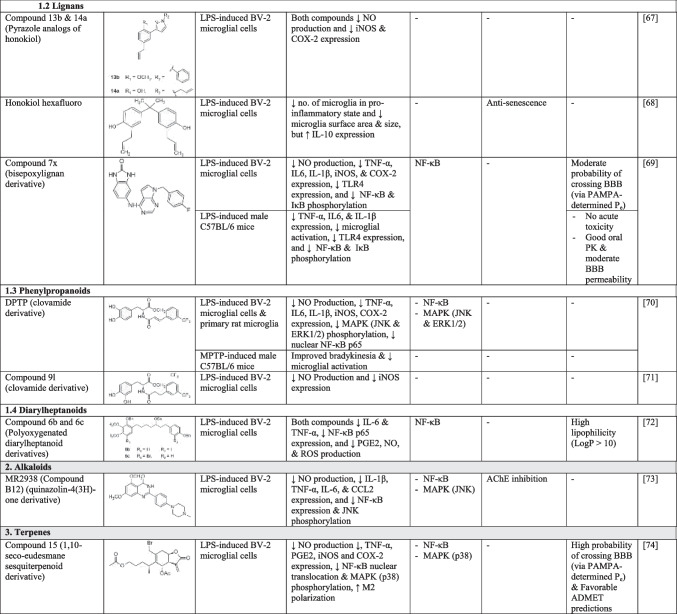
Studies involving the discovery of anti-neuroinflammatory compounds based on the modification of natural compounds

*AChE* acetylcholinesterase, *ADMET* absorption, distribution, metabolism, excretion, and toxicity, *BBB* blood–brain barrier, *CM* conditioned medium, *LPS* lipopolysaccharide, *PAMPA* parallel artificial membrane permeability assay, *P*_*e*_ permeability coefficient

*Polyphenols* are a large group of plant secondary metabolites with diverse biological effects; however, for AD and other neurodegenerative diseases, they are primarily regarded for their antioxidant and anti-inflammatory activities. In addition, several compounds from this group, such as resveratrol, quercetin, icariin, and curcumin, have been shown to have neuroprotective effects in either in vitro or in vivo studies [75]. For this reason, numerous studies have tried to improve upon many polyphenolic structures, especially in terms of increasing lipophilicity, so that they may pass through the BBB and increase their biological activities.

As summarized in Table 1, the modifications of polyphenols through aromatic rings substitution, esterification, or halogenation can lead to derivatives with potent anti-neuroinflammatory activities. This is demonstrated by their abilities to inhibit the expression of proinflammatory mediators and enzymes (e.g., TNF-α, IL-1β, IL-6, iNOS, and COX-2). In cases where relevant signaling pathways were further investigated, it was discovered that these compounds mostly exert their effects by modulating inflammatory signaling pathways such as NF-κB and MAPK. For example, 3′-O-(3-chloropivaloyl) quercetin (CPQ), a quercetin pivaloyl ester, was shown to inhibit NO production. In LPS-induced BV-2 microglial cells, it also suppressed the expression of proinflammatory mediators (e.g., TNF-α and iNOS) and prevented the nuclear translocation of NF-κB. These effects seemed to be more potent compared to the original compound, quercetin. This derivative also exhibited high lipophilicity [62].

Beyond their anti-neuroinflammatory effects, some of these compounds demonstrated multifunctional activities. Compound a8, a 7-O-1, 2, 3-triazole hesperetin derivative, inhibited the expression of TNF-α, IL-1β, iNOS, and COX-2 and prevented the phosphorylation of NF-κB p65 subunit. It also appeared to be an AChEI, an Aβ aggregation inhibitor, and a metal chelator, potentially offering therapeutic benefits for addressing the multifactorial AD. In animal studies, compound a8 improved long-term memory in scopolamine-induced mice, as evidenced by decreased escape latency and increased time in the target quadrant, and more frequent platform crossing in the Morris Water Maze test [65].

*Alkaloids* are a group of secondary metabolites containing nitrogen atoms in their structure and are associated with several biological activities [47]. In a recent study, MR2938 (B12), a quinazolin-4(3*H*)-one derivative with 4-piperazine phenyl ring substitution, was shown to inhibit NO production. It also suppressed the expression of proinflammatory mediators (IL-1β, TNF-α, IL-6, and CCL2) in LPS-induced BV-2 microglial cells by inhibiting NF-κB and MAPK signaling pathways. Furthermore, MR2938 was identified as a noncompetitive inhibitor of AChE, exhibiting a docking pore similar to that of donepezil with a half maximal inhibitory concentration (IC_50_) value of 5.04 μM [73].

*Terpenes* are formed from two or more isoprene units and have been reported to have anti-neuroinflammatory activities in both in vitro and in vivo models [47]. Compound 15, a 1,10-seco-eudesmane sesquiterpenoid derivative with a bromine substitution, inhibited NO production with an IC_50_ of 0.7 μM. Furthermore, it suppressed the expression of iNOS, COX-2, TNF-α, and PGE2 by inhibiting NF-κB and MAPK signaling pathways. Compound 15 also increased the level of Arg1, suggesting a role in regulating microglia polarization. Results from the parallel artificial membrane permeability assay (PAMPA) suggested that the compound had a high permeability coefficient (*P*_e_) [74].

These studies can be further categorized based on compound groups into polyphenols (e.g., flavonoids, lignans, phenylpropanoids, and diarylheptanoids), alkaloids, and terpenes. In general, it seems that structural modifications such as substitution, esterification, and either nitrogenation or halogenation appear to improve the biological activity of these compounds. In some cases, the modified compounds exhibited greater activity than the parent compounds. Additionally, studies that investigated absorption, distribution, metabolism, excretion, and toxicity (ADMET) properties or membrane permeability found that the modified compounds often demonstrated high lipophilicity or high *P*_e_, suggesting the potential to cross the BBB.

#### Rational Drug Design

Rational drug design refers to the development of drug molecules based on the desired properties for a specific drug target [76]. The first step in this approach is to identify and characterize drug targets, which are biomolecules integral to disease development (e.g., receptors and enzymes). The critical aspect of this process is determining the target’s three-dimensional (3D) structure, including its binding site, using technology such as X-ray crystallography or nuclear magnetic resonance (NMR). This structural information enables the design of molecules that can bind to the target with high affinity. By understanding the interactions between the target and its endogenous ligands, researchers can design compounds that mimic or enhance these interactions, resulting in high binding affinity. This approach is called structure-based drug design (SBDD) [76, 77].

On the other hand, if the 3D structure of the drug target is unknown, ligand-based drug design (LBDD) can be used. This approach focuses on studying the structure and physicochemical properties of known ligands associated with the target of interest, allowing researchers to develop new drug molecules with similar biological activities [78]. In addition, the use of LBDD can also facilitate the identification of pharmacophores or chemical structures that are known to have biological activities [77].

In the following section, studies on the discovery of anti-neuroinflammatory compounds are categorized into three groups based on their structure design strategies: (1) compounds developed using a pharmacophore with previously reported activities, (2) compounds designed based on endogenous ligands, and (3) compounds obtained through structure conjugation. The key findings from these studies are then briefly discussed. The details of each study could be found in Table 2.

Firstly, drug design using pharmacophores is a well-established strategy to discover new drugs. As seen in Table 2, three studies utilized various known pharmacophores to develop anti-inflammatory compounds based on their previously reported activities. For example, carboline is a heterocyclic structure with many biological activities, and compounds containing either α- or β-carboline in the structure have been reported to modulate many of AD pathologies, including neuroinflammation. In the present study, three series of α-carboline analogues were synthesized and evaluated for their anti-neuroinflammatory properties. Compound 5 d, identified as the best performing compound, demonstrated significant NO inhibition in the screening assay. This compound was found to inhibit inflammatory enzymes such as iNOS and COX-2. In addition, compound 5 d decreased the phosphorylation of both NF-κB p65 subunit and IκBα, resulting in the inhibition of NF-κB signaling pathway. It exhibited drug-like properties, including good absorption and good permeability across the BBB. In an animal model, compound 5 d effectively prevented LPS-induced aberrations in the zebrafish model [79]. The other studies in this group, which focused on antidepressant fluoxetine and the pharmacophore quinoline, also followed similar design principles [80, 81].

**Table 2:**
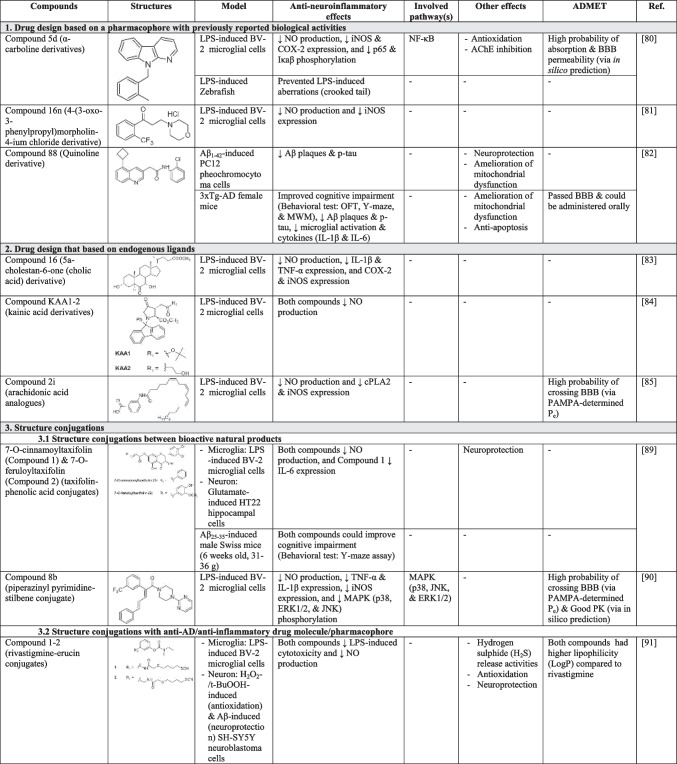

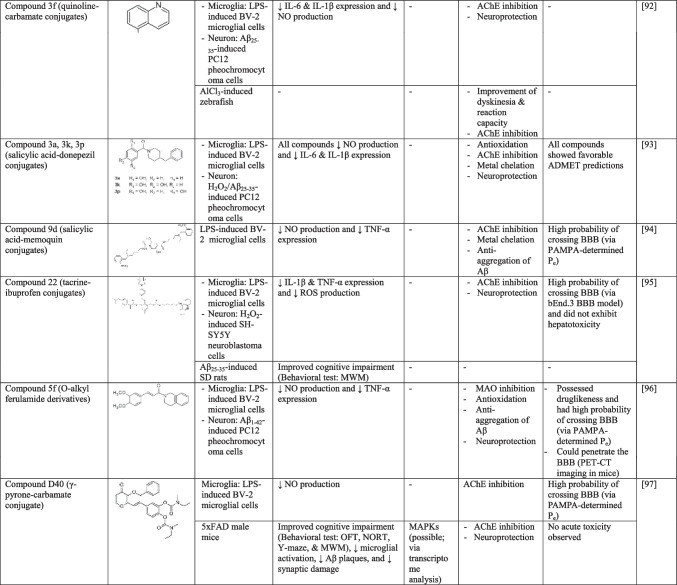
Studies involving the discovery of anti-neuroinflammatory compounds based on rational drug design

*Aβ* beta amyloid, *AChE* acetylcholinesterase, *ADMET* absorption, distribution, metabolism, excretion, and toxicity, *BBB* blood–brain barrier, *LPS* lipopolysaccharide, *MAO* monoamine oxidase, *MWM* Morris water maze test, *NORT* novel object recognition test, *OFT* open field test, *PAMPA* parallel artificial membrane permeability assay, *P*_*e*_ permeability coefficient

Secondly, drug design based on endogenous ligands, or the use of such ligands as a template for the synthesis of new compounds, can also be classified as the LBDD approach. Three studies with this approach have been identified [82–84]. Cholic acid, a bile acid synthesized in the liver from cholesterol, has been reported to exhibit many biological activities, including inflammatory modulation [85]. Kainic acid, an analog of the excitatory neurotransmitter glutamate, has been shown to modify cell morphology and stimulate the secretion of inflammation-related mediators in microglia [83]. Arachidonic acid, a product of phospholipases A_2_ (PLA_2_) enzymes, serves as a precursor of eicosanoids and prostanoids, key mediators involved in inflammatory processes [84].

In all three studies, analogs derived from these endogenous compounds were found to inhibit NO production and suppress the expression of various proinflammatory mediators. Moreover, compound 2i, an arachidonic acid analog, demonstrated inhibitory activity against cytosolic PLA_2_ (cPLA_2_) and exhibited high *P*_e_, indicating its high probability of crossing BBB [82–84].

Lastly, structure conjugation is a part of the drug design strategy that aims to address multiple pathologies of a disease with a single drug molecule, an approach known as “Multitargeted-directed ligands (MTDLs).” In this strategy, pharmacophores with different known biological activities are combined into one molecule with multiple activities. Compared to the traditional “*one molecule, one target*” approach, MTDLs offer several advantages, such as enhanced therapeutic efficacy, more predictable pharmacokinetics and pharmacodynamics, and greater suitability for treating complex multifactorial diseases like cardiovascular diseases or neurodegenerative diseases. In addition, this approach is also more beneficial compared to the use of combination regimen for the treatment of a disease, as it could mitigate potential adverse events (drug interactions and/or toxicities) arising from polypharmacy [86].

There are several strategies for obtaining MTDLs that depend on the similarities between the pharmacophore and/or scaffolds, as well as the size of the drug target’s binding pocket [87]. In the present work, eight publications concerning structure conjugation were identified. These publications were further categorized into two groups: the conjugation of natural bioactive compounds with each other, and the conjugation of compounds where at least one is an existing anti-AD drug, an anti-inflammatory drug, or both.

For the first group of MTDLs, or the conjugations of natural bioactive compounds with each other, the approach is similar to that of modifying natural compounds, as it builds on previous reports of their anti-neuroinflammatory effects. A total of two publications were included in this group [88, 89]. For example, a study of conjugates between taxifolin (a flavonoid compound) and either cinnamic acid or ferulic acid, both previously discovered to have neuroprotective activities, found that the new compounds have anti-neuroinflammatory and antioxidant activity in vitro, and reversed cognitive impairment induced by Aβ_25–35_ in Swiss mice [88]. In another study, the conjugate between the piperazinyl pyrimidine and stilbene (compound 8b) was found to inhibit proinflammatory mediators (TNF-α, IL-1β, and iNOS) through MAPK pathway inhibition. Additionally, the compound showed high BBB permeability but low solubility, which is a potential point for further optimization [89].

The second group of MTDLs includes compounds in which at least one compound or pharmacophore is an established anti-AD or anti-inflammatory drug. This approach is based on the hypothesis that the resulting conjugated molecule should retain the biological activities of both parent compounds. Among the six publications reviewed, the anti-AD drugs used include rivastigmine, donepezil, and tacrine, all of which are AChEIs. The anti-inflammatory drugs include ibuprofen and salicylic acid. The remaining components used for conjugation are either natural compounds (such as sulforaphane/erucin and ferulic acid) or bioactive pharmacophore (such as quinoline and propargyl group) [90–96].

One notable aspect of the structure conjugation approach is that most studies aimed to investigate more than one type of anti-AD activities, the so-called MTDL approach. Besides anti-neuroinflammation, which is the focus of this work, the potentials for AChE inhibition, neuroprotection, metal chelation, and anti-Aβ aggregation of the compounds were also explored. Particularly, AChE inhibition seems to be another major mechanism of interest due to the long-standing cholinergic hypothesis and the current anti-AD regimen. According to the MTDL strategies described by Li et al. [87], all studies reviewed fall under either the non-cleavable linked pharmacophore [90–93] or the cleavable linked pharmacophore [94]. The main advantage of the linked pharmacophore strategy is that both pharmacophores can recognize and bind to their respective targets. Furthermore, if the binding pocket is narrow, such as that of AChE, a flexible linker can be used to distance the two pharmacophores, allowing the appropriate one to access the binding site without restriction. In the case of a cleavable-linked pharmacophore, the two molecules can be released and act independently in vivo [87]. For instance, in the work by Liu et al., the synthesized compound was designed to undergo ester hydrolysis under an oxidative environment, thereby releasing tacrine and ibuprofen derivatives [94].

#### Screening Approach

One integral discipline in drug discovery is drug screening, in which molecules from a library of compounds are rudimentarily tested using a well-established protocol, either biochemical or cell-based, to find molecules with desirable biological properties. Screening with increasing rate, quantity, or automation can be termed “high-throughput screening (HTS),” though the distinctions between the two are not very clear [97]. Unlike the rational drug design discussed above, screening offers advantages in situations where the drug target is not yet well characterized [97, 98].

In the present work, five studies have been identified using a screening approach for the discovery of anti-neuroinflammatory compounds. These studies, along with their key findings, are summarized in Table 3. Briefly, several compounds and structures with no previously reported anti-neuroinflammatory activities were tested in vitro, specifically using microglial cells (primary or immortalized cell lines) as the model. In line with other studies from previous approaches, the synthesized compounds demonstrated the ability to inhibit the expression of proinflammatory mediators in LPS-induced microglial cell models. Further investigations in some studies revealed that they acted through the inhibition of either MAPK or NF-κB signaling pathways, while some explored different anti-AD effects, such as neuroprotection or AChE inhibition [99–103].

**Table 3:**
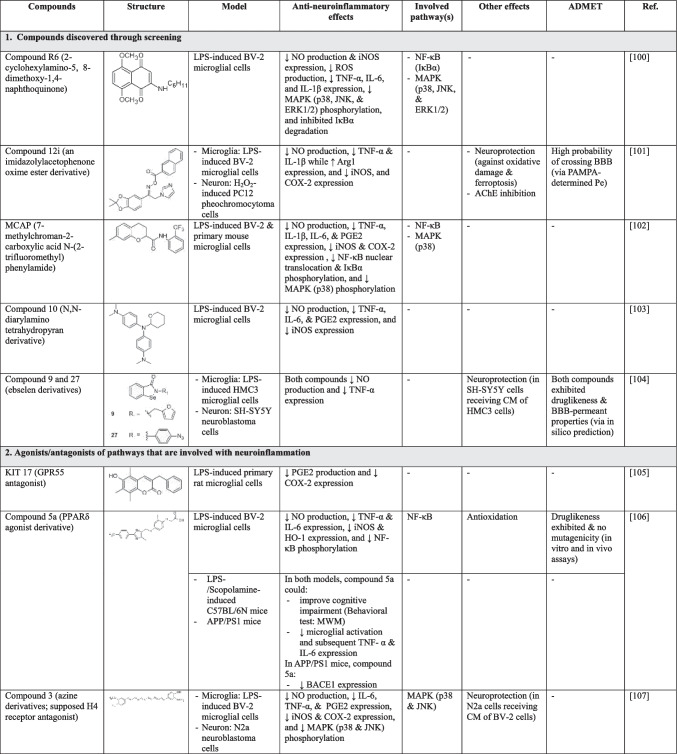

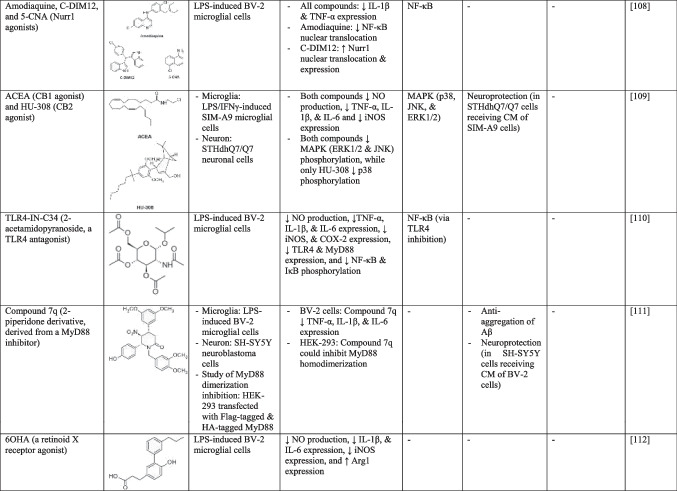
Studies involving the discovery of anti-neuroinflammatory compounds based on the screening approach

*Aβ* beta amyloid, *AChE* acetylcholinesterase, *ADMET* absorption, distribution, metabolism, excretion, and toxicity, *BBB* blood–brain barrier, *CM* conditioned medium, *LPS* lipopolysaccharide, *PAMPA* parallel artificial membrane permeability assay, *P*_*e*_ permeability coefficient

Although the main pathways involved in neuroinflammation and microglial activation have been discussed in the introduction section, it is important to note that numerous other receptors and enzymes have also been implicated in inflammatory signaling pathways, even though their specific involvement remains incompletely understood. Therefore, modulating these drug targets via their known ligands may contribute to the attenuation of neuroinflammation. As summarized in Table 3, this review covers seven studies that investigated the anti-neuroinflammatory activity of compounds targeting the following receptors and signaling pathways: TLR4, MyD88, Cannabinoid 1 and 2 receptor (CB_1_R and CB_2_R), G protein–coupled receptor 55 (GPR55), nuclear receptor–related 1 (Nurr1), histamine 4 (H4) receptor, PPAR delta (PPARδ), and retinoid X receptor (RXR) [104–111].

TLR4 and MyD88, as previously mentioned, are the upstream components of NF-κB, a signaling pathway which plays a major role in neuroinflammation. The endocannabinoid system and its related receptors, including GPR55, are a class of G protein–coupled receptors that were found to be relevant in AD pathologies. Particularly, CB_2_R, which is primarily expressed in microglia, was discovered to be overexpressed in the brains of both AD mouse model and AD patients compared to the control group. In addition, CB_2_R activation was shown to inhibit the release of proinflammatory cytokines and enhance the Aβ phagocytic activity of microglia [112–114]. Activation of the H4 receptor was found to be involved in other inflammatory diseases [106]. And lastly, Nurr1, PPARs, and RXR belong to the nuclear receptor (NR) superfamily, ligand-activated transcription factors that can promote gene expression upon dimerization with another NR. In AD, they seem to share quite similar roles. That is, they could inhibit the activation of NF-κB while promoting the expression of antioxidant enzymes, resulting in overall anti-neuroinflammatory effects. Nonetheless, the roles of these receptors are still not fully elucidated; endogenous ligands of Nurr1 are yet to be identified, while determining the exact roles of RXR is complicated due to their obligate dimerization with other types of NR, including PPARs [105, 115–117].

From this brief introduction, these receptors and adaptors can be categorized into two types: those whose activation promotes neuroinflammation (TLR4, MyD88, and H4R) and those whose activation contributes to the attenuation of neuroinflammation (CB_1_R/CB_2_R, GPR55, Nurr1, PPARδ, and RXR). According to the studies included in this review, the use of antagonists (or inverse agonists) for the former case and agonists for the latter resulted in a reduction in the expression of proinflammatory mediators in in vitro models. Additionally, the signaling pathways related to their antagonism or agonism were confirmed and further elucidated. As such, discovering ligands that can modulate these pathways might be another feasible approach for the discovery of novel anti-neuroinflammatory compounds.

#### Drug Repurposing

Drug repurposing refers to a strategy to find new uses for approved drugs that are unrelated to their original indication. This strategy has many advantages over traditional drug discovery, including already established drug safety, reduced development time, and lower investment costs. The discovery of new pharmacological activities of a drug can be achieved through various methods, such as retrospective clinical analysis (from reported adverse effects), compound screening, or pharmacological analysis [118].

For this work, two studies involving a drug repurposing approach were identified, both of which are concerned with telmisartan, an angiotensin II receptor blocker (ARB), as detailed in Table 4 [119, 120]. The angiotensin II receptor is a part of renin–angiotensin system (RAS), which plays an important role in the regulation of cardiovascular physiology [121]. Chronic activation of RAS is generally associated with raised blood pressure and contributes to the development of hypertension. However, recent evidence suggests that RAS is also involved in neuroinflammation and dementia pathogenesis. Specifically, activation of type 1 angiotensin II receptor (AT_1_R) in the brain has been shown to induce vasoconstriction, increase the production of ROS, enhance inflammation, and promote Aβ production. In contrast, activation of type 2 angiotensin II receptor (AT_2_R) resulted in promoting vasodilation and reducing inflammation [121, 122].

**Table 4:**
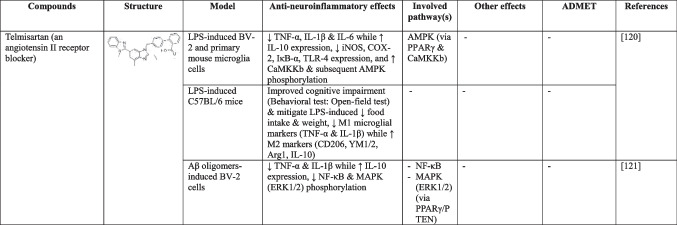
Studies involving the discovery of anti-neuroinflammatory compounds based on the drug repurposing approach

*Aβ* beta amyloid, *ADMET* absorption, distribution, metabolism, excretion, and toxicity, *LPS* lipopolysaccharide

Previously, several studies had investigated the role of ARBs on neurological cognition in murine models and found that, besides blocking AT_1_R, ARBs seem to exert anti-inflammatory effects through the activation of PPAR gamma (PPARγ) [120]. Both included studies intended to elucidate the underlying mechanisms of telmisartan in neuroinflammation. Collectively, the findings confirmed that telmisartan mitigated neuroinflammatory responses induced by LPS and Aβ oligomers. This was observed by a reduction in the expression of proinflammatory mediators (such as TNF-α, IL-1β, and IL-6), as well as by the promotion of microglia into M2 polarization (as evidenced by the increase in M2 markers). In addition, telmisartan was shown to reduce the activation of NF-κB pathway.

Further analysis using pathway-specific antagonists confirmed that telmisartan exerts its effects through AMPK and PPARγ signaling pathways. Moreover, telmisartan could reduce M1 markers and increase M2 markers in the brain of LPS-induced mouse models. It could also reverse negative effects, such as reduced food intake, weight gain, and anxiety in the mice. According to these results, telmisartan could be further explored as a potential anti-neuroinflammatory agent [119, 120].

### Discussions of Future Perspectives

Neuroinflammation is now recognized as a key mechanism in the development and progression of AD. It has been established to have intertwining roles in the exacerbation of other important AD pathologies, including Aβ deposition, NFT formation, and neuronal loss. Currently, there are no approved anti-AD drugs that specifically target neuroinflammation. However, several such drugs are currently undergoing clinical trials, and many others are in the process of drug discovery and development. This work aimed to review the publications in the last decade (2014–2025) that focus on the investigation of anti-neuroinflammatory activity of compounds derived from chemical synthesis and structural modification to understand the current trends of anti-neuroinflammatory AD drug discovery, particularly in terms of drug targets (including pathways, receptors, and enzymes), the sources of investigated structures, and other effects besides anti-neuroinflammation.

From 46 relevant publications, the compounds investigated in these articles could be categorized based on the main approaches to their discovery (as detailed in their respective section) into four groups: the modification of natural compounds, rational drug design, drug screening approach, and drug repurposing approach. In addition, these publications focus on investigating at least one of the three major inflammation-related signaling pathways: NF-κB, MAPK, and AMPK. While it is possible that this work might not actually include every publication on such topic due to the gap in search terms, a few observations could still be made in terms of the current trends and future directions of anti-neuroinflammatory compounds for AD treatment.

#### Modification of Natural Compounds is the Most Promising Approach of Anti-Neuroinflammatory Compound Discovery

Out of 46 publications, the first approach, modification of natural compounds in this category, stands out as the most prevalent approach in anti-neuroinflammatory compound discovery. This might be because many natural products already have a large amount of available literature regarding their biological activities. Therefore, the goal of this approach is to introduce functional groups or atoms that may enhance the biological activity or pharmacokinetics of the original compound. The main strategies employed are substitutions on aromatic rings, esterification, or introduction of nitrogen or halogen atoms. These modifications either result in increased lipophilicity, which could improve their pharmacokinetics, or increased steric effects and/or electronegativity which could improve the binding or reactivity of the molecules. Despite these general observations, there are also some exceptions. For example, in the study by Mateeva et al., the chalcone compound with methoxy substitutions exhibited greater NO inhibitory activity than the one with halogen substitutions [60].

Various natural compounds, such as resveratrol, curcumin, or quercetin, have been investigated for the treatment of AD in either preclinical or clinical studies [123–125]. However, despite showing promising in vitro results, their failures to demonstrate efficacy, especially in clinical trials, may stem from their poor bioavailability or inability to effectively cross the BBB [125, 126]. Therefore, improving such compounds by means of structural modification is still a promising approach to anti-neuroinflammatory compound discovery.

#### Multitarget-Directed Ligands (MTDLs) Approach as the Future of Anti-Alzheimer’s Agent Discovery

Based on the studies included, there appears to be growing interest in the development of MTDLs for the treatment of AD. At least 12 publications have evaluated compounds for multiple pharmacological activities besides anti-neuroinflammation, including AChE inhibition, anti-Aβ aggregation, neuroprotection, and metal chelation. This approach is the most prevalent in the group involving structural conjugation, as the primary objective of such an approach is to discover a molecule with several anti-AD activities. However, some works in other groups also aimed to assess multiple anti-AD effects of the synthesized compounds in a similar manner.

Besides antioxidant activity, which is intrinsically connected to anti-inflammatory activity, neuroprotection appears to be one of the alternative activities that were most investigated. Generally, its methodology could be categorized into two approaches. Some studies opted to introduce stressors such as Aβ or H_2_O_2_ to the neuronal cells directly to study the neuroprotective effect of the tested compounds. In contrast, other studies attempted to simulate microglia and neuron crosstalk, either employing microglia and neuron co-culture using the Transwell system or transferring the CM from anti-neuroinflammatory compound-treated microglia to neuronal cells. These two approaches seem to serve different purposes: the former focused on the ability of the compounds to directy negate stressor-induced toxicity, while the latter approaches assess the effects of attenuated microglia on neuronal survival. Considering the complexity of glial-cell interactions in the actual CNS microenvironment [127], the use of microglia-neuron model might be better suited for the investigation of anti-neuroinflammatory compounds in the context of neurodegenerative diseases.

Other commonly investigated activities, which include AChE inhibition and anti-Aβ aggregation, are all in accordance with previously established hypotheses of AD, namely cholinergic hypothesis and amyloid hypothesis. While correcting ACh imbalance does not directly address the underlying AD pathologies, it still has short-terms benefit in terms of cognitive improvements. In addition, in light of the recent investigation into the α7 nicotinic receptor (α7 nAChR) and their role in AD pathogeneses, anti-AChE activity might prove to be synergistic with anti-neuroinflammatory activity [128]. Similarly, even if previous studies on anti-Aβ therapeutics did not yield positive results, they might prove beneficial in combination with compounds targeting other pathologies [44].

In addition, although the neuroinflammation hypothesis is probably the most promising hypothesis at the moment, it has also been suggested that the search for AD treatment should take on a more holistic approach [42]. In other words, since age is the most established risk factor of AD, it might be more helpful to view AD as the culmination of various physiological changes brought about by aging, e.g., mitochondrial dysfunction, calcium dysregulation, DNA damages, and abnormal autophagy, and try to address them all simultaneously [42, 129]. While this is easier said than done, owing to the limited understanding of true nature of AD, some publications included here attempted to address some of them. In the work by Sasia et al. (2024), the honokiol hexafluoro was also investigated for its ability to ameliorate cellular senescence in LPS-induced BV-2 microglial cells [68]. Similarly, other studies attempted to elucidate the metal chelation properties of the synthesized compounds [65, 92, 93]. With the increasing recognition of AD as a multifactorial disease, the MTDL approach will surely become more widely adopted in the future; however, on which combination of activities would be the most effective for AD treatment still requires further investigation.

#### The Investigation of Drug-Like Properties and Blood–Brain Barrier (BBB) Permeability of Anti-Neuroinflammatory Compounds

As mentioned previously, the investigation of drug-like properties and BBB permeability are especially important for the anti-neuroinflammatory compounds; however, based on the included publications, only some studies reported in silico ADMET and BBB permeability predictions of their synthesized compounds. Even more scarce is either the in vitro or in vivo evaluation of such properties. While in vivo models are cost-extensive and in vitro models do suffer from various limitations that make extrapolating the results to human challenging [51, 54], they are still good starting points to determine the pharmacokinetics of drug candidates. And if conducting them is not possible for various reasons, in silico predictions nowadays are greatly accessible with either free-of-charge online application services such as SwissADME (http://www.swissadme.ch/) or paid programs such as the commercial version of Biovia Discovery Studio Visualizer. These softwares certainly could not replace actual experiments and evaluations; however, they could serve as complementary screening tools, particularly in the early phase of the drug discovery pipeline.

## Conclusion

To summarize, chronic microglial activation has been implicated in several neurodegenerative diseases, and the modulation of inflammation-related pathways like NF-κB, MAPKs, and AMPK is being explored as a strategy to discover disease-modifying therapeutics. In the past decade, the neuroinflammation hypothesis of AD has gained widespread attention and greatly influenced the field of its drug discovery and development. This review has included summaries of recent publications (between 2014 and 2025) involving the discovery of anti-neuroinflammatory compounds derived from chemical synthesis in preclinical models. In addition, these publications could be categorized based on their main approach to drug discovery into four groups. Like drug discovery in other diseases, the modification of natural compounds appears to be the most common approach. Despite this, there is also a specific approach, MTDL, that intends to address the multifactorial nature of AD. Besides anti-neuroinflammation, most studies still focused on previously established activities like anti-AChE or anti-Aβ; however, with better understanding of contributing factors like aging in AD and increased identification of potential drug targets, other combinations of activities are also being explored, and more research is required to find the combinations that are effective in AD. Lastly, as CNS-acting agents, these anti-neuroinflammatory compounds must penetrate BBB to exert their effects. As such, it is suggested that determination of such properties, either via in silico predictions or permeability assays, should be carried out during the preliminary screening process.

## Data Availability

No datasets were generated or analyzed during the current study.

## Abbreviations

AChE: Acetylcholinesterase
AChEI: Acetylcholinesterase inhibitor
AD: Alzheimer’s disease
ADMET: Absorption, distribution, excretion, metabolism, and toxicity
AMPK: Adenosine monophosphate-activated protein kinase
APP: Amyloid precursor protein
ARB: Angiotensin receptor blocker
Arg1: Arginase 1
Aβ: Beta amyloid
BBB: Blood–brain barrier
CCL2: CC chemokine ligand 2
CNS: Central nervous system
COX-2: Cyclooxygenase-2
CM: Conditioned medium
cPLA_2_: Cytosolic Phospholipase A2
GPR55: G-protein-coupled receptor 55
IFN: Interferon
IC_50_: Half-maximal inhibitory concentration
IκB: Inhibitor of κB
IL: Interleukin
iNOS: Inducible nitric oxide synthase
LBDD: Ligand-based drug design
LP: Ligand-based drug design
LPS: Lipopolysaccharide
MAO: Monoamine oxidase
MAPK: Mitogen-activated protein kinase
MTDL: Multitarget-directed ligand
MW: Molecular weight
MWM: Morris water maze
NFT: Neurofibrillary tangle
NF-κB: Nuclear factor-κB
NR: Nuclear receptor
NO: Nitric oxide
NOS: Nitric oxide synthase
Nrf2: Nuclear factor erythroid 2–related factor 2
NSAID: Nonsteroidal anti-inflammatory drug
PAMPA: Parallel artificial membrane permeability assay
P_e_: Permeability coefficient
PK: Pharmacokinetics
PPAR: Peroxisome proliferator–activated receptor
ROS: Reactive oxygen species
SAR: Structure–activity relationship
TNF-α: Tumor necrosis factor-α
